# P-168. Antimicrobial Resistance Patterns at a Private Referral Hospital in Blantyre, Malawi

**DOI:** 10.1093/ofid/ofae631.373

**Published:** 2025-01-29

**Authors:** Matthew Cappiello, Lindsay S Lim, Tadala Rambiki, Kalkidan Chala Dosso, Freedom Felix Mandala, Juan Infante, Eugene W Liu

**Affiliations:** Loma Linda University Medical Center, Loma Linda, California; University of Alabama at Birmingham, AdventHealth, Orlando, Florida; Blantyre Adventist Hospital, Blantyre, Blantyre, Malawi; Blantyre Adventist Hospital, Blantyre, Blantyre, Malawi; Malamulo Adventist Hospital, Makwasa, Thyolo, Malawi; University of California Riverside, Riverside, California; Loma Linda University, Loma Linda, California

## Abstract

**Background:**

Antimicrobial resistance (AMR) is expected to be a global health security risk in Africa in coming decades. While AMR data is available from Malawi public hospitals, data from Malawi’s burgeoning private hospital sector is limited.

Initial Hospital Antibiogram Data, Fall 2021 - Spring 2022

**Figure:**
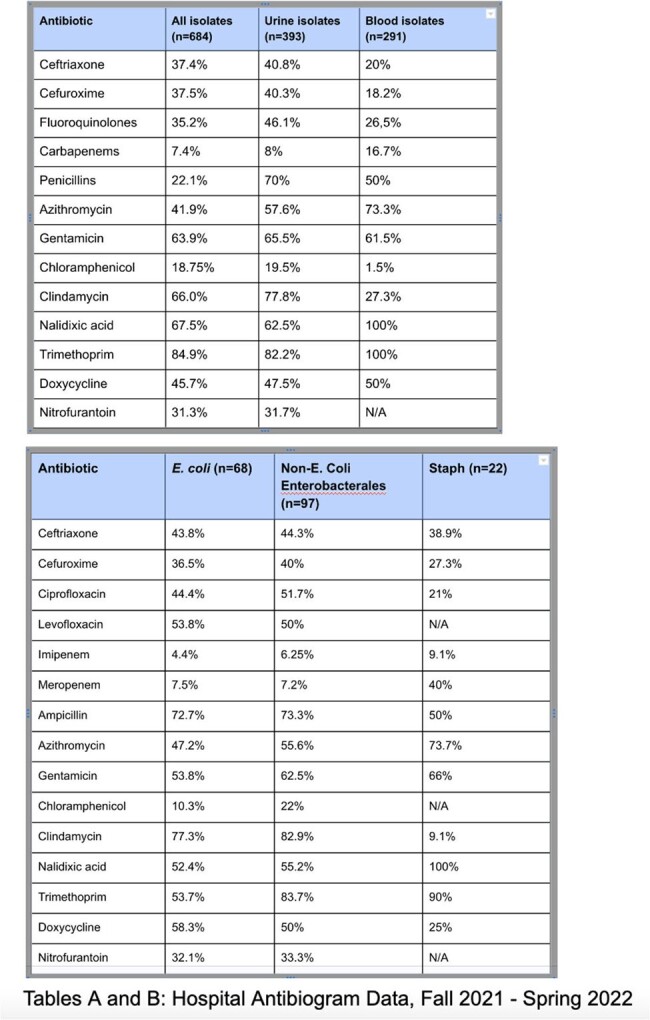

**Methods:**

An antibiogram was obtained for clinical blood and urine cultures from October 2021 through April 2022 at a private referral hospital in southern Malawi (n=684). Logistic regression was used to assess for patient-related determinants of AMR, as well as effects on patient outcomes.

Tests of Association for AMR Determinants

**Figure:**
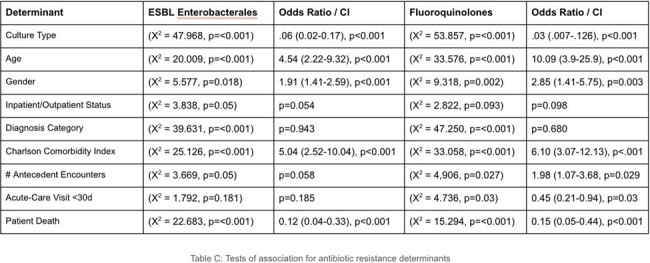

**Results:**

High AMR rates were seen in *E. coli* (37.4% ceftriaxone, 35.8% ciprofloxacin, 32.5% trimethoprim), as well as unexpected carbapenem resistance (7.4% of tested isolates). Statistically significant tests of association linked both fluoroquinolone (X^2^ = 4.91, p=0.027) and third-generation cephalosporin resistance (X^2^ = 3.67, p=0.05) to patients who had increased numbers of antecedent hospital encounters. Fluoroquinolone resistance was associated with increased risk for acute-care follow up within 30 days of antibiotic prescription (X^2^ = 4.73, p=0.03). Third-generation cephalosporin resistance (suggestive of ESBL production) showed association with older age (X^2^ = 20.009, p=< 0.001) and female gender (X^2^ = 5.58, p=0.018) A multiple logistic regression model examining variables associated with ceftriaxone-resistant Enterobacterales showed increased risk of patient death (OR 5.99, CI 1.37–26.17, p=0.017), as well as higher Charlson comorbidity index (OR 1.14, CI 1.02-1.28, p=0.024).

Over 40% of patients with culture-positive results were treated empirically, without follow up for review of sensitivities. Choice of empiric antibiotic differed from national guidelines, with only 32% UTI patients and 8.4% pharyngitis patients receiving recommended first-line regimens. Local point-prevalence data sometimes appeared to be discordant with national data. As one example, all three recommended cystitis regimens on national formulary guidelines displayed >=30% resistance rates on local antibiogram data, including ciprofloxacin, nitrofurantoin, and cefuroxime.

Logistic Regression for Determinants of ESBL Enterobacterales

**Figure:**
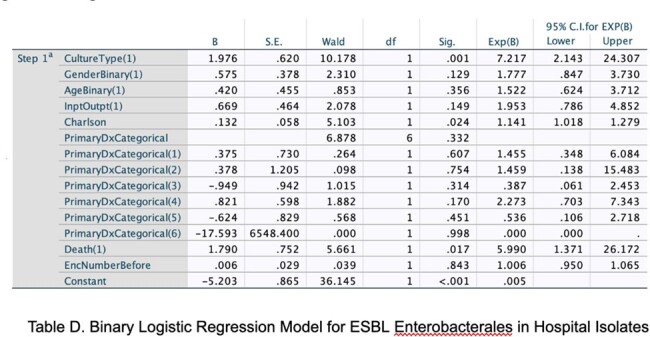

Multiple logistic regression model

**Conclusion:**

Further research needs to assess best practices for stewardship in regional private hospitals, as well as determinants for AMR that encompass markers of healthcare utilization.

Demographic Descriptive Statistics

**Figure:**
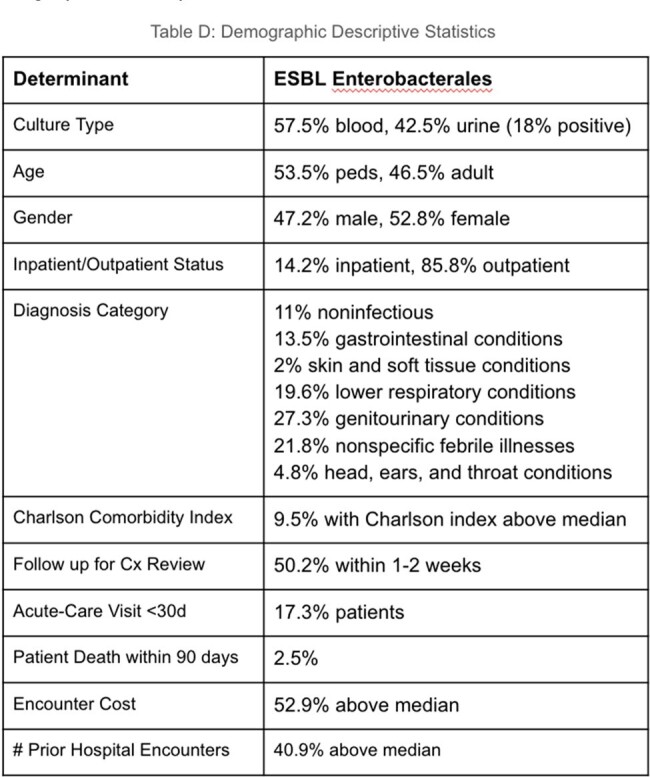

**Disclosures:**

**All Authors**: No reported disclosures

